# All hands on deck during the COVID-19 pandemic. Maintaining face-to-face medical education and clinical placements

**DOI:** 10.1371/journal.pone.0306129

**Published:** 2024-07-01

**Authors:** Hilary Humphreys, Ronan Baxter, Judith Gilroy, Gianpiero L. Cavalleri, Tom O’Connor, Steve W. Kerrigan, Fidelma Fitzpatrick, Aoife Gilligan Quinn, Sam McConkey, Kilian McGrogan

**Affiliations:** 1 Department of Clinical Microbiology, Royal College of Surgeons in Ireland University of |Medicine and Health Sciences, Dublin, Ireland; 2 Estate and Support Services, Royal College of Surgeons in Ireland University of |Medicine and Health Sciences, Dublin, Ireland; 3 Student, Academic and Regulatory Affairs, Royal College of Surgeons in Ireland University of |Medicine and Health Sciences, Dublin, Ireland; 4 School of Pharmacy and Biomolecular Science, Royal College of Surgeons in Ireland University of |Medicine and Health Sciences, Dublin, Ireland; 5 School of Nursing and Midwifery, Royal College of Surgeons in Ireland, Dublin University of |Medicine and Health Sciences, Dublin, Ireland; 6 Department of Microbiology, Beaumont Hospital, Dublin, Ireland; 7 Centre for Mastery: Personal, Professional and Academic Success, Royal College of Surgeons in Ireland, Dublin University of |Medicine and Health Sciences, Dublin, Ireland; 8 International Health and Tropical Medicine, Royal College of Surgeons in Ireland University of |Medicine and Health Sciences, Dublin, Ireland; 9 Clinical Directorate of Medicine, Beaumont Hospital, Dublin, Ireland; 10 Mercer’s Medical Centre, Royal College of Surgeons in Ireland University of |Medicine and Health Sciences, Dublin, Ireland; Xiamen University - Malaysia Campus: Xiamen University - Malaysia, MALAYSIA

## Abstract

Medical students must have robust educational experiences, graduate and commence timely employment. Here, we describe how the Royal College of Surgeons in Ireland (RCSI) delivered clinical placements in medical programmes over the first three waves of the COVID-19 pandemic in Ireland, including a student-centred, holistic approach to students’ educational, social and health needs with strong student involvement, re-organising the university’s primary care team, developing COVID-19 PCR testing on site and re-focusing communications and student services. This resulted in re-arranging the students into learning communities, and students and staff electronically recorded their COVID-19 symptom status daily. In-person observed structured clinical examination and other clinical exams progressed. No lockdown of any campus occurred. Over the two senior years, 693 students completed 15,000 weeks of clinical and experiential learning across 104 sites, similar to previous years, including anatomy practicals, procedural skills training, simulated ward rounds and patient encounters, case-based presentations and small group tutorials. The compliance rate with the daily symptom tracker was 91%. The percentage response rate and the number of students providing feedback from October 2020 to April 2021 was as high as 50%. The overall response rate was 33%. By mid-May, 93–95% of students in the two senior years had had at least one dose of the SARS-CoV-2 vaccine, with 99% fully vaccinated by the start of the next academic year in autumn 2021. Over the period of testing for SARS-CoV-2, just over 22,000 samples were processed, of which 0.79% were positive; no medical student acquired COVID-19 or was associated with nosocomial transmission. The total investment by the RCSI in Dublin, was €9.3m (€1.2 in capital expenditure and €8.1 in operational expenses). Continuing face-to-face clinical placements during a pandemic was possible through a multi-model approach that prioritised two-way communication, compliance with national public health advice and student screening.

## Background

The unprecedented and ongoing Coronavirus disease 2019 (COVID-19) pandemic was challenging given the transmissibility of the virus in the community and amongst patients and healthcare workers (HCW). In the healthcare setting, HCW became ill and could potentially transmit the virus to patients and colleagues. Furthermore, infected staff and staff close contacts had to remain off work in many instances for 10–14 days, which had implications for staffing levels [1]. The rate of infection amongst HCW during outbreaks was up to 20% and regular and repeat testing of HCW was recommended as part of infection prevention and control (IPC) measures during suspected outbreaks [2, 3].

Medical students are the doctors of tomorrow, but the delivery of medical education during the pandemic was challenging [4]. Sustaining face-to-face teaching, especially clinical placements, was difficult. Some medical schools cancelled activities and moved content online [4–7]. Many assessments did not take place; in 32 of 33 UK medical schools, final year students reported that 38% of objective structured clinical examinations exams (OSCE) had been cancelled [8]. Medical students need to graduate and fill important healthcare roles, but there are significant implications from moving entirely on-line such as generalised anxiety, depression and reduced physical activity [9–11].

During the pandemic, it was essential to prioritise safety while maximising core educational activity. A continuous ‘pipeline’ of medical graduates is needed to replenish the health service, replacing continuously retiring or departing staff. The priority was to ensure that students had a robust educational experience, became competent safe graduates, and stepped into employment on time.

The first case of COVID-19 in Ireland was confirmed on February 29^th^ 2020. Schools and colleges closed and large gatherings were cancelled on 12 March 2020. By 24th March 2020, almost all businesses were shut and a national mandatory stay at home order was implemented on March 28^th^, followed by a phased easing of restrictions from mid-May. Schools re-opened in September, but in response to a 14-day incidence rate of 120 per 100,000 population, another country-wide lockdown occurred (excluding schools) in October 2020. Restrictions were eased in early December 2020 and following an additional surge in late December, a new state-wide lockdown occurred which this time included schools.

Healthcare vaccination commenced in Ireland on 29^th^ December 2020, rapidly followed by vaccination of residents of long-term care facilities in late January 2021. Schools reopened in March 2021. Higher education institutions were issued with governmental sector wide guidance, which allowed essential in-person activities to continue, within strict and limited criteria.

Here we outline how the RCSI addressed the challenges presented by the pandemic. The objectives of the RCSI approach were to ensure the safety of students, staff and patients, and deliver clinical placements and face-to-face teaching sessions. This was achieved by providing effective two-way communication with students, undertaking student screening and finally, ensuring student compliance with public health measures. In describing how we responded to the pandemic, we reflect on what measures were introduced, their relative success and the implications if a similar pandemic occurred in the future, in the hope that this may help in guiding other medical schools faced with equivalent circumstances.

## Methods

### Population

The participants were the medical student population in the RCSI University of Medicine and Health Sciences during the pandemic, i.e. from 2020 to 2022 inclusive. Staff in the university were participants as part of a multi-disciplinary team to develop measures to mitigate the effects of the pandemic and in monitoring their effectiveness.

### Outcomes measures

The effectiveness of the interventions designed to manage the impact of COVID-19 on student health and learning were assessed in a number of ways:

- Compliance rates with daily health checks. Anonymised data from the online systems were used to collect these.
- Anonymised written student feedback.
- SARS-CoV2 positivity rates collected from our onsite testing.
- Vaccination rates.
- Student participation and attainment data, including completion of clinical rotations, classroom and tutorial sessions within the time period.

Generally, the data extend up to the end of semester for the academic year 2020–21, i.e. approximately mid-May, even if there was some variation as to when the year ended for some students, e.g. earlier compared to later years. Furthermore, some national data were incomplete or delayed due to a cyberattack on the Irish Health Service Executive (HSE) databases in the spring of 2021.

### Interventions

**Fig 1:**
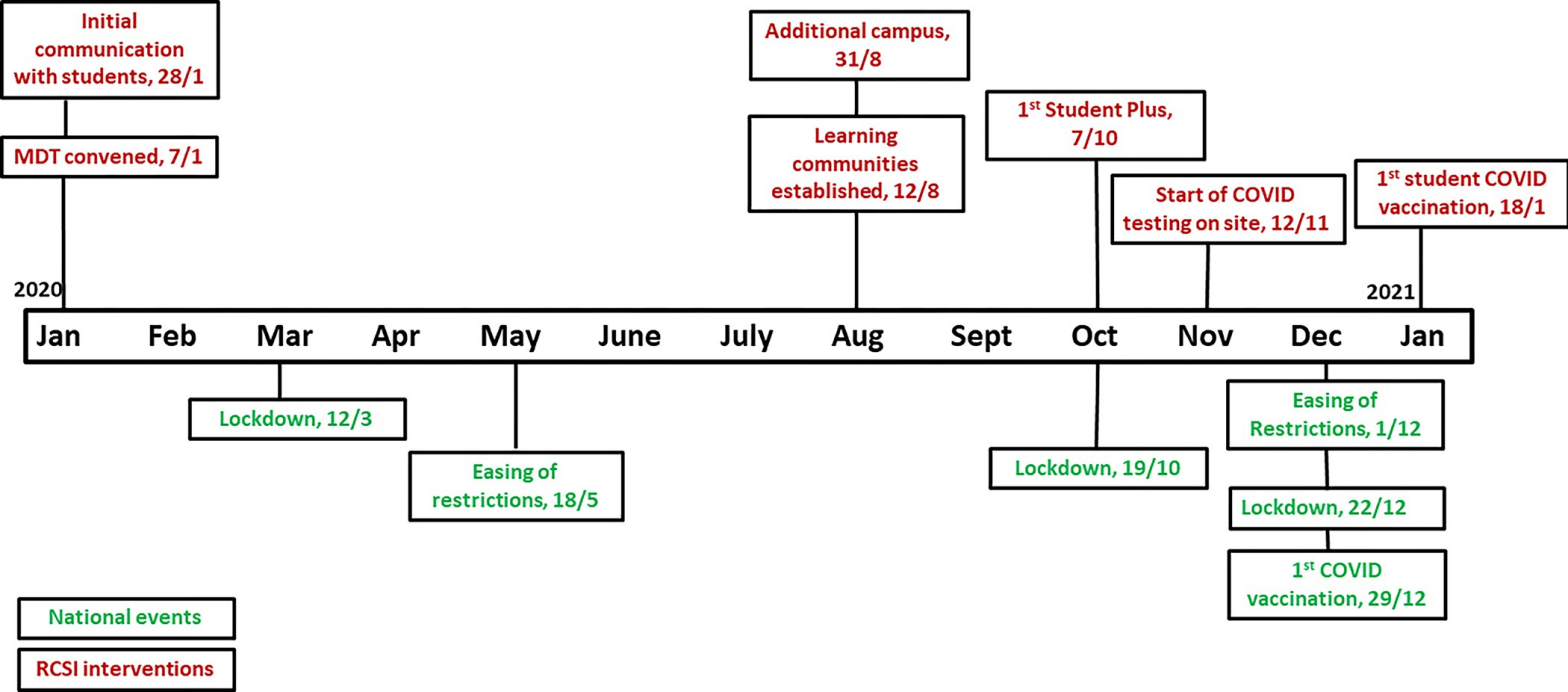
Measures taken by the RCSI to protect students, staff and patients while delivering medical education during the COVID-19 pandemic and interventions at national level in Ireland, 2020–2021.

An outline of the interventions together with those introduced nationally, e.g. lockdowns and restrictions on the movement of the public is provided in Fig 1.

Those interventions against which the outcomes were measured were the development of a multi-disciplinary team (MDT), enhanced engagement with students including regular student surveys and and the development of a COVID-19 information hub, setting up an online system for daily symptom recording amongst students, the establishment of student testing on site, providing an additional campus with closed student learning communities to facilitate social distancing, student vaccination and contact tracing of COVID-19 positive students, and liaison with public health authorities.

### Initial actions–Spring/summer 2020

The Royal College of Surgeons in Ireland (RCSI) is an international University of Medicine & Health Sciences that graduates students in medicine (undergraduate and graduate entry), pharmacy and physiotherapy, is the largest provider of postgraduate training in nursing in Ireland, undertakes biomedical research & provides surgical training. Excluding trainees in surgery and radiology, there are currently approximately 4,600 students of which about 3,000 are full-time. The student body is internationally diverse with 82% of medical students travelling to Ireland from over 67 countries. Students mainly live in the centre of Dublin in self-contained rented housing, and not in university halls of residence.

**Fig 2:**
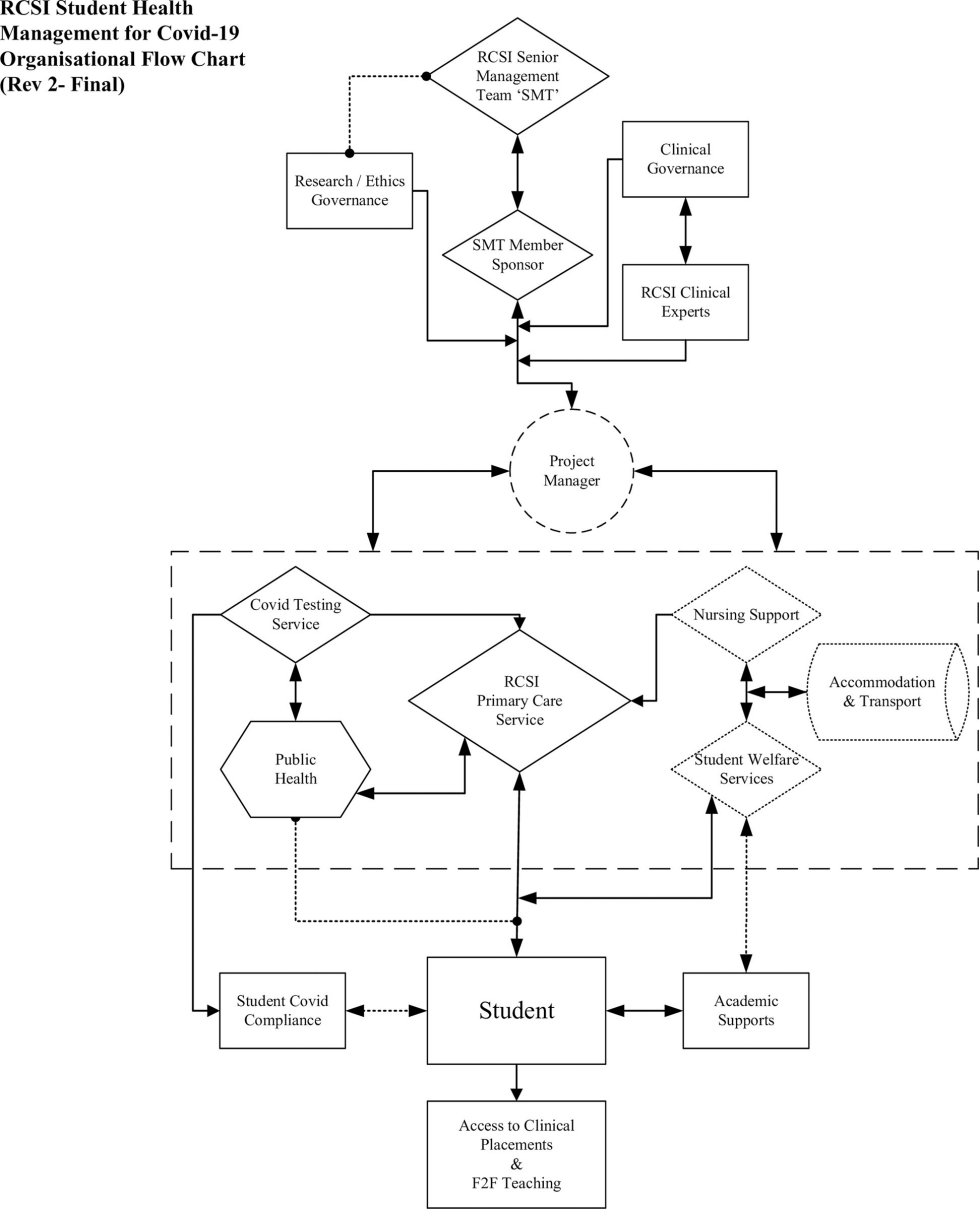
RCSI student health management for COVID-19. Organisational flow chart.

In response to the COVID-19 pandemic, the university developed a holistic co-ordinated approach to students’ educational, social and health needs, which was co-designed with students, and delivered by a MDT that built on existing strengths. (Fig 2). This MDT was led by a member of the senior management team, who called on expertise and insights from colleagues at every level across professional services, i.e. finance, academic affairs including registry, careers and wellbeing, student services including gym and university societies, estates and campus security, travel department, human resources, and communications. Clinical colleagues managing student health directly through the RCSI primary care student health centre, colleagues from the School of Nursing & Midwifery who provided support to students testing positive, those with considerable expertise in clinical microbiology and infectious diseases with access to national policy makers, and scientists with expertise in molecular testing protocols, were also included. This MDT, often including up to 20 individuals, met online three times a week and more at the height of the pandemic. Support for the work of the MDT was evident by the attendance and contributions of the Vice Chancellor (VC) of the University at the majority of these meetings.

Engagement with the students was through the students union, class representatives and regular “class calls” on MS Teams. University wide messages were delivered through a variety of channels including a newsletter from the Deputy Vice Chancellor for Academic Affairs (initially daily and then weekly) and regular video addresses from the VC streamed through MS Teams.

Student feedback and comments were collected through the “RCSI Student Pulse”, a monthly anonymous survey of students’ experience. The survey was based on a random sample of a third of the RCSI student population and participation was voluntary. The data was collected between October 2020 and April 2021 by the RCSI Quality Enhancement Office. The poll asked three questions to a randomly selected sample of students, approximately one third of the undergraduate student population each time. The purpose of the poll was to provide quick feedback to facilitate rapid changes at the time, to highlight good practice and to indicate matters of concern that could be actioned to enhance student experience.

Students replied by typing into a free text box. The questions asked were:

Q1. Please briefly outline the main aspects of your RCSI experience that worked well during the last four weeks.

Q2. Please briefly outline the things about your RCSI experience that could be improved.

Q3. Please use the space below to provide any additional comments for the following RCSI facilities and services.

Categorization into positive or negative feedback was made from the comments provided to Q1 or Q2 and the sentiments expressed across all three questions, e.g. “I did feel communication was lacking” would be categorised as “negative”, while “They have always been very fast in their replies and supportive”, would be considered positive.

In March 2020, we anteponed final medical clinical examinations to avoid the first COVID-19 wave in hospitals. All other students were asked to return to their countries/ homes, prioritising students graduating in late spring 2020. A website with detailed information and a COVID-19 information hub were established. A student census was undertaken to ensure that all students had returned safely to their family homes, and teaching, and clinical sessions were innovatively converted online.

### Planning for autumn 2020 and beyond

Over the summer of 2020, we planned how to return our international students to Ireland safely through re-organising the university’s primary care team, establishing COVID-19 testing on site, and re-focussing communications and student services. This was to simulate a normal medical school experience (including face-to-face sessions), and care for and, support students far from home, family and close friends, when Ireland’s COVID-19 rates rapidly oscillated between the lowest to the highest in Europe [12].

In September 2020, to facilitate socially distanced face-to-face classes, the city centre campus was reserved for Year 1 students. An additional campus at a convention centre in a major sports stadium was secured for Year 2 and 3 students, with students in Year 4 and 5 being based at clinical sites.

When returning in August to Dublin, students were provided with a framework of activities, encompassing all aspects of university life. International students were required to pre-book an RCSI pick-up at the airport & were transported to their accommodation, where they were supported while restricting their movements for the mandated 14 days. This included the delivery of a pre-built MacBook to every new student to ensure appropriate access to the RCSI virtual learning environment and online assessments systems, and scheduled social contacts from an academic and pastoral learning community lead who arranged, ‘virtual coffees’. Between days 7 to 10 after arrival, we arranged COVID-19 screening, which largely anticipated subsequent recommendations on testing students [13].

We sought to maintain face-to-face encounters and clinical placements, whilst limiting possible transmission by organising students into ‘learning communities’, i.e. small closed groups of students always scheduled together, and cycling these communities through the campus for HyFlex teaching [14–16], clinical placement, and library access. Students practised 2-metre social distancing and mask wearing at all times on the campus. Day-to-day collaboration with students and staff, and using new teaching tools like open-mic sessions, and recorded lectures, ensured a smooth autumn semester. We succeeded through project- based co-ordination of efforts from non-clinical supports such as academic affairs, student services, training and finance, and clinical services such as primary care, clinical liaison and testing, while maintaining strong relationships with affiliated clinical sites (Fig 2).

### Infection prevention and control measures, including testing

We sought to minimise the acquisition of COVID-19 amongst our student population through a combination of IPC measures (e.g. social distancing, mandatory masking wearing while on campus, enhanced cleaning and decontamination protocols and hand hygiene), complying with public health advice, testing for SARS-CoV-2, prompt isolation of positive cases, active contact tracing, and movement restrictions on student close contacts. Based on Irish public health advice and in conjunction with local public health specialists, a simple Microsoft Teams form was created, that required students to check their symptoms daily if they had been in close contact with a case, or had travelled from overseas. The form was co-designed with students, and student representatives advised on how to encourage peer compliance. A daily “invite” was sent to class groups, weekly statistics on completion were shared with the class representatives and regular, personal follow up with students reporting symptoms or non-completion, ensued. All COVID-19 clinical issues amongst the student body, including symptom assessment and advice, referral, contact concerns, liaison with public health and decision making on return to placement timings, were dealt with by our in-house comprehensive primary care team. This provided free seven days a week care to all students.

From January 2021, RCSI medical students on clinical rotations were included in the national programme of healthcare vaccination, which was delivered via our clinical sites.

We established an on-campus testing facility in August 2020 under supervision of the primary care service with clinical operational assistance for swabbing being provided by the School of Nursing and nurses from the Department of Surgery. This facility mirrored the standards and protocols of public health testing facilities in Ireland [17]. Initially PCR testing of nasopharyngeal swabs (NPS) was performed offsite. In November 2020, we established an onsite COVID-19 laboratory, facilitating faster test turnaround times, and tighter integration of testing with the university’s contact tracing and IPC programme. Having confirmed the efficacy of saliva samples compared to nasopharyngeal swabs, we started to process these at the start of the next academic year to detect SARS-Co-V2 [18]. RNA extraction from NPS samples was performed on a KingFisher™ Flex instrument using 200 μL of NPS sample input and a MagMax Viral/Pathogen II Nucleic Acid Isolation Kit, as per manufacturers’ instructions, and in line with the CDC protocol [19].

Before the January 2021 semester started, all students (regardless of overseas travel) were tested for COVID-19 at the RCSI testing facility. We introduced regular and frequent COVID-19 screening of clinical students by PCR, initially based on varying levels of endemicity, or each time a student changed a clinical placement. This occurred approximately every two weeks, with approximately 500 tests weekly from November 2020.

When positive cases were detected, RCSI student health teams initiated rapid contact tracing internally across the RCSI, as well as referring the student to public health for contact tracing in the wider community. During early 2021, when public health contact tracing was not possible nationally due to the massive case numbers, we continued to contact trace and test close contacts of all students. Where a student tested positive, they received a daily ‘phone call from clinical staff to monitor their health, and a separate contact from student welfare and support services, to assist their general health and wellbeing. Changes in their academic programme were arranged as necessary, including waiving the need to complete applications for exceptional circumstances, to allay anxiety relating to assessment impacted by reported infection or isolation.

### Social and mental health supports

To reduce the risk of student infection through travel or subsequent on-campus outbreaks, our international students were discouraged from returning home to celebrate the winter holiday season at the end of 2020. However, we employed an additional student welfare officer who started in June 2020, in recognition of the increasing complexity and need in the area of mental health and student wellbeing. We also supported students through activities such as virtual drop-in coffee breaks, online fitness classes and mindfulness sessions.

Mindful that this might be the first time that many students were away during the holiday (i.e. December 2020-January 2021), we supported their mental health and well-being through a programme of festive activities, compliant with public health guidelines with limited social gatherings. Students received Christmas hampers, including baked goods made by staff, and students delivered Christmas bouquets to older people in the local community. Such was that commitment, the RCSI was shortlisted for The Times higher award for Outstanding Support for Students (https://twitter.com/rcsi_irl/status/1438524331185770508).

### Ethics and consent to participate

“All hands on deck during the COVID-19 pandemic. Maintaining face-to-face medical education and clinical placements” falls into the category of audit, and as such, did not require RCSI Research Ethics Committee (REC) approval.” The REC consequently agreed that individual consent was not therefore required. However, students and staff were fully informed and kept up to date about the measures taken and why they were necessary. i.e. to ensure their safety, and in the face of an unprecedented public health emergency.

## Results

The overarching RCSI mission during the pandemic was to ensure that students continued their education, that their health and wellbeing as well as that of staff and patients were protected, that there was no interruption in the supply of medical graduates to continue to provide health services, that medical and other staff continued to contribute to the local and national response, and finally that research, including on SARS-CoV-2, continued. This commitment was evident from the huge effort of all staff and led by the ongoing MDT. The total investment by the RCSI in control measures in Dublin, including testing (see below) was €9.3m, compromising €1.2 in capital expenditure and €8.1 in operational expenses.

### Compliance with daily health checks

**Fig 3:**
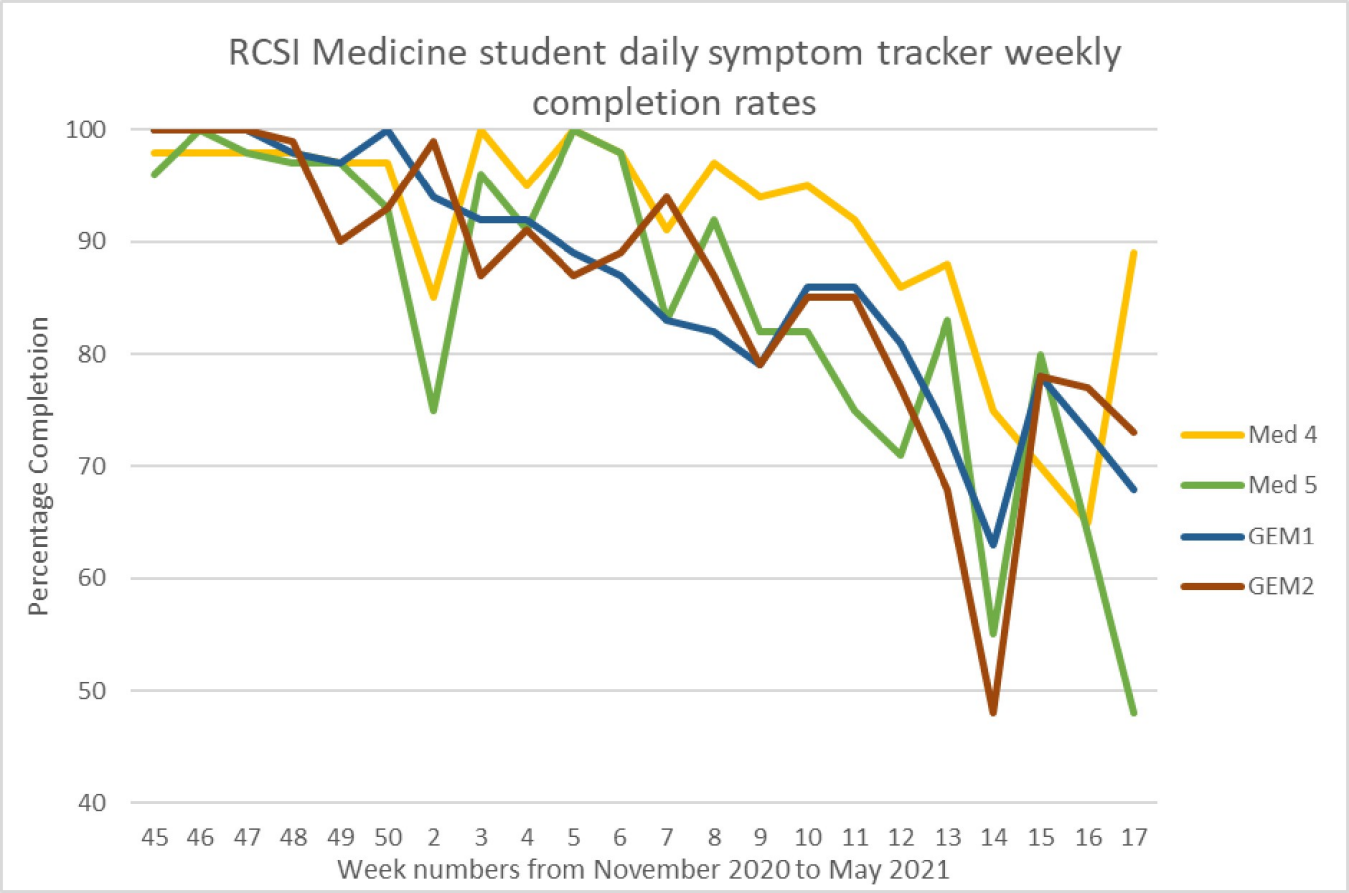
Completion of daily online questionnaire about possible symptoms of COVID-19 (Med, undergraduate medical school year; GEM, graduate entry medicine year).

The compliance rate across direct and graduate entry medicine was 91% over the course of the academic year. Regular, multi-channel, two-way communication with and involvement of students resulted in high adherence to daily health checks, even if these fell somewhat towards the end of the academic year (Fig 3).

### Student feedback

Feedback from students was general positive, with most welcoming the regular screening and supportive interventions, even if the response rate fell over time. The percentage response rate and the number of students (in parentheses) providing feedback for October, November 2020, January, February, March and April 2021 were, 50% (415), 43% (362), 34% (288), 29% (238), 23% (187), and 20% (163), respectively. The overall response rate was 33%.

Many missed the social interactions and suggestions for improvement largely related to access to vaccine, particularly in the latter half of the post-Christmas semester. Counselling referrals for mental health support increased by 23% in 2020 compared to 2019 and self-referral rates increased by 64% over the same period. However, higher levels of self-awareness and self-care, and a greater awareness of the services may in part explain the latter.

There was a lot of effort put in at university level into the responses to students as feedback and providing reassurance were a priority. For example, a “CEO’s video message” went out to students and was linked to the feedback received. In these, negative issues or concerns previously raised were addressed and where possible relevant information provided, as requested by the students. There were also additional communications by email.

**Fig 4:**
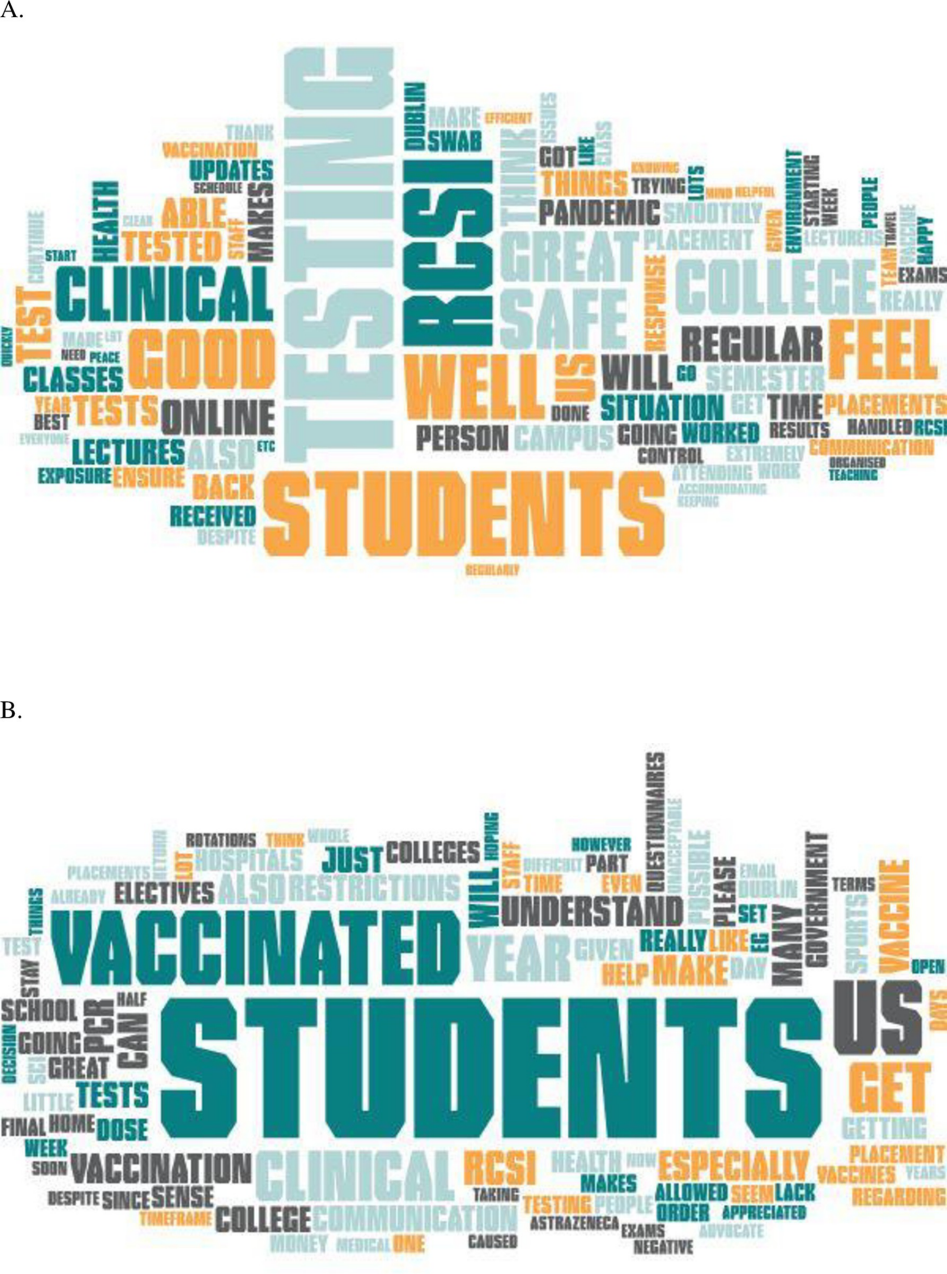
Word clouds of A. positive feedback from medical students with the word COVID removed. B suggestions for improvement from medical students with the word COVID removed.

Fig 4 outlines some key themes from the feedback in the form of word clouds. This was achieved by conducting a sentiment analysis based on the free texts as described [20]. Many of the prominent texts indicated positive terms such as well, safe, good and great.

### Vaccination

**Fig 5:**
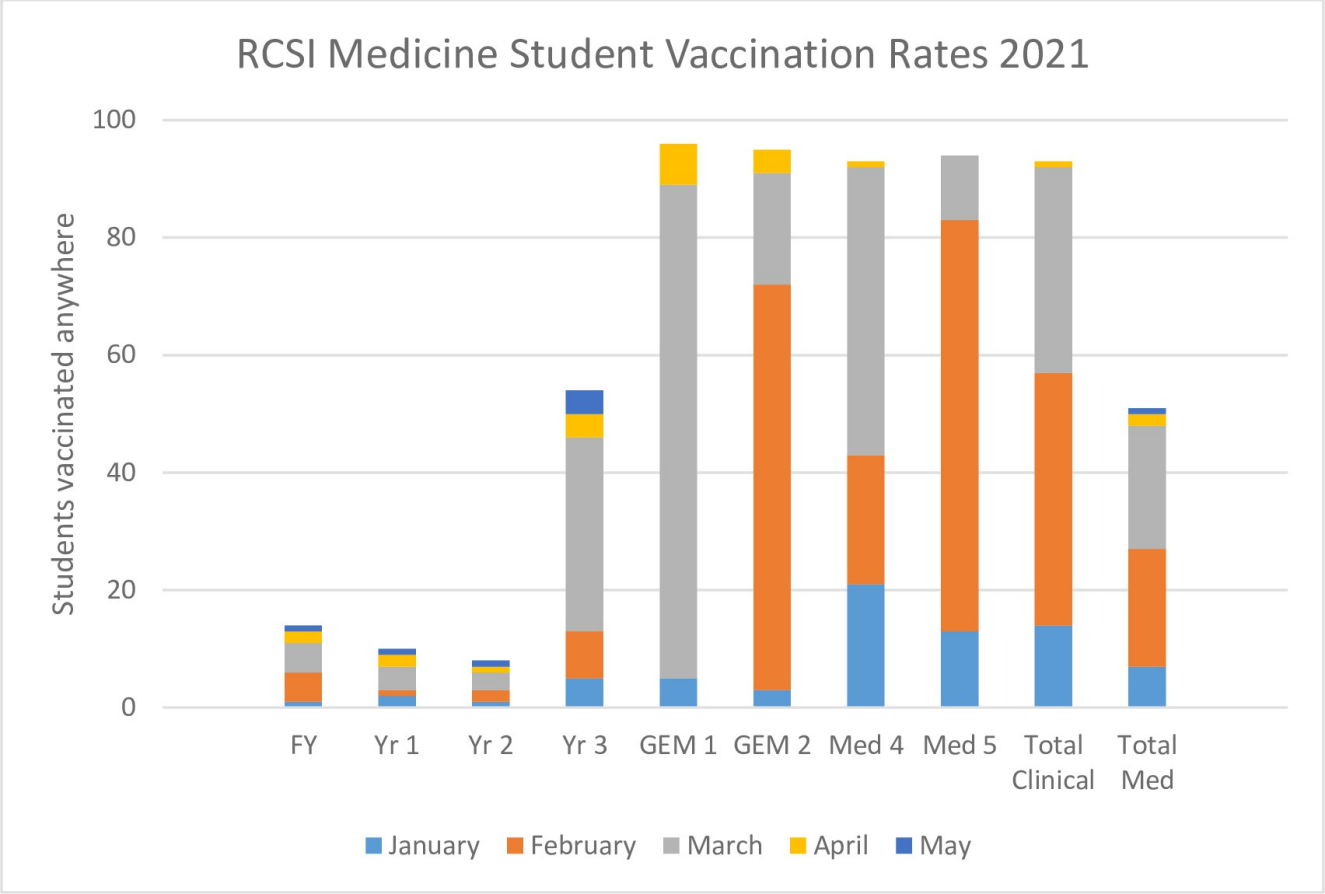
RCSI medical student vaccination rates, 2021 (FY, foundation year; Yr1, Year 1 of undergraduate medicine, etc., GEM graduate entry medicine).

Vaccination of healthcare students on clinical sites was prioritised by Irish health authorities from the onset of availability, and medical student vaccination was delivered by hospitals and other clinical sites. By the middle of June 2021, 64% of medical students had been able to secure a vaccine, but for students in the clinical years or attending a scheduled clinical attachment, this rose to 95% of students who had been fully or partially vaccinated. (Fig 5). By the start of the next academic year, i.e. 2021–22, 99% were fully vaccinated.

### Testing for SARS-CoV-2

**Fig 6:**
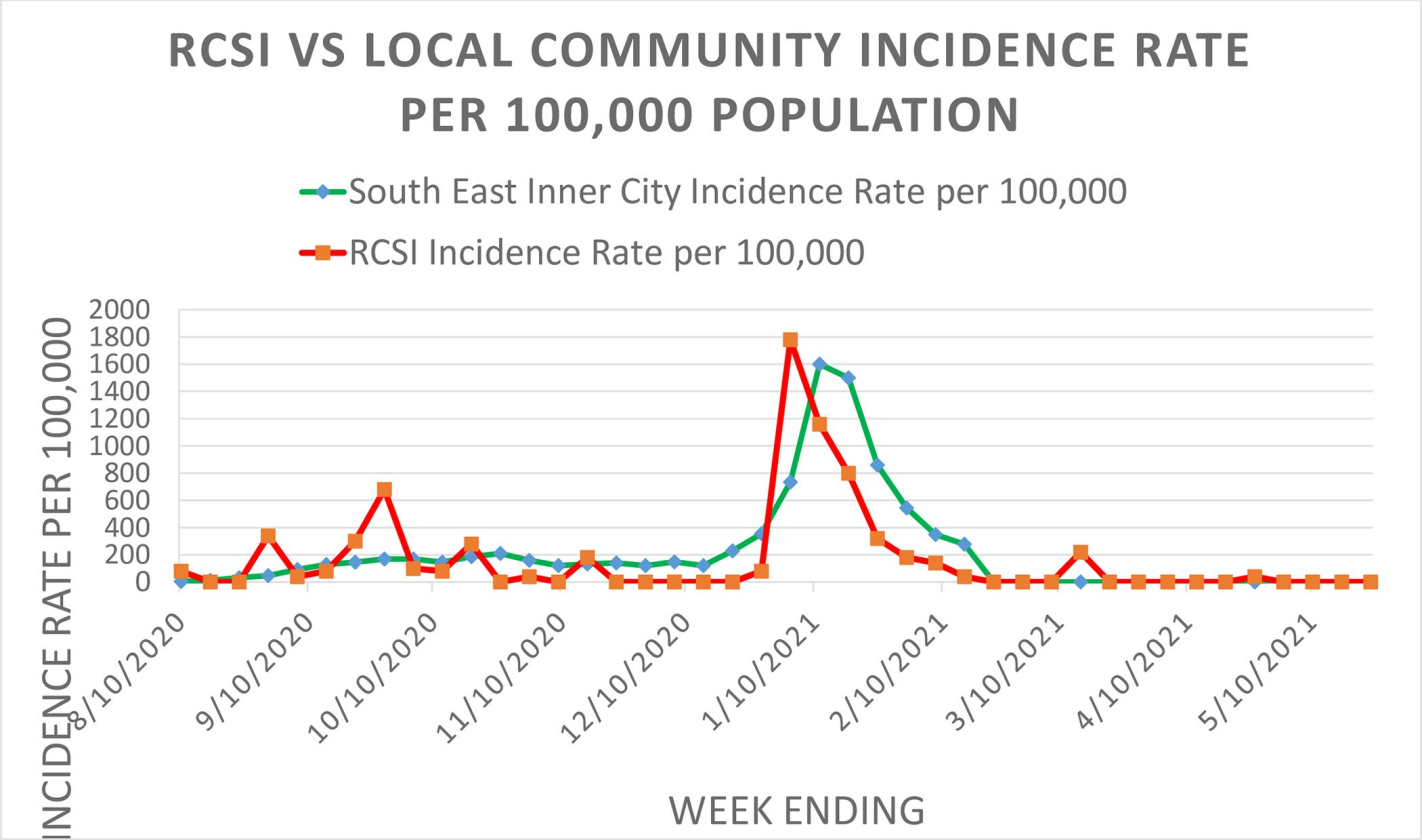
RCSI *versus* local community incidence of COVID-19 rate per 100,000 population.

We initiated our in-house SARS-CoV2 laboratory in November 2020, although students had been tested since August, using an external laboratory. The total expenditure for testing was €550,000, including €240,000 in staff costs and €220,000 for laboratory consumables. Over the period of testing, just over 22,000 samples were processed, of which 0.79% were positive [21]. The rate of testing and associated positive cases and a comparison with the local population from the HSE between August 2020 and May 2021 (inclusive) are shown in Fig 6. Genotyping indicated that the SARS-CoV-2 variants mirrored those in the community [21].

### Clinical and experiential learning

We ensured that 693 students in the two senior years completed nearly 15,000 weeks of clinical and experimental learning, across 104 sites, from 28^th^ September 2020 to 1^st^ April 2021, with access dipping in January and February 2021, coinciding with the third national surge in Ireland. This was similar to previous years before the pandemic. Students throughout the medical school attended thousands of hours of in-person anatomy practicals, clinical and procedural skills training, case-based sessions and small group tutorials. Innovative simulated ward rounds and patient interactions were delivered on one hospital site and on the main university campus, and with these and other learning outcomes met the equivalent regulatory requirements. Hence, the student learning experience was as equivalent as it could be, given that this all occurred during a pandemic.

The numbers graduating before and during the pandemic were similar, i.e. 314, 321, 334 and 351 in 2018/9, 2019/20, 2020/21 and 2021/22, respectively. The percentage of students that passed the final medical examinations were 98.1%, 98.2%, 98.6% and 98.6% in 2019, 2020, 2021 and 2022, respectively. The percentage that obtained honours (2^nd^ and 1^st^ class) were 64%, 79.7%, 70.8% and 71.2% in 2019, 2020, 2021 and 2022, respectively.

Ongoing testing with investigations of clusters and outbreaks in hospitals indicated that no medical student acquired infection during any placements or was a source of nosocomial transmission. Furthermore, no lockdown of any campus facilities was required.

## Discussion

While the pandemic called on huge reserves of resilience and adaptability amongst medical students, their educators and others, our strong view is that medical education involves the acquisition of clinical skills that cannot be fully delivered via distance learning, notwithstanding the huge efforts that others and we made. Hence, face-to-face sessions were deemed essential and desirable. However, some medical schools came up with novel solutions to address various aspects of the curriculum, including online learning through a simulated experience covering primary care and surgery [22–26]. Shin and colleagues developed a virtual case-based general surgery clerkship curriculum in Ohio, USA [24]. It covered common surgical conditions such as acute cholecystitis, and knowledge scores increased but the authors acknowledge that a significant limitation was the absence of operating theatre experience such as participating in team dynamics and scrubbing [24]. In London, UK, medical educators piloted placements for 16 students which included rotations in clinical care, research and audit, nursing care and compassionate care. The students responded positively, for example, they valued working collaboratively rather than competitively, and felt that their knowledge of COVID-19 increased and their concerns decreased [25]. Many of the interventions in all these centers were well received by students with feedback indicating increased knowledge and insight, but the student response was before and after the specific initiative, rather than through a comparison with cohorts of students in previous years. In addition, often the number of students involved in the initiatives was relatively small and or addressed some but not all aspects of clinical training [22, 24–27]. An interesting perspective from one of our students was that due to the pandemic, some non-urgent patients were not seen, such as in gynaecology, resulting in less exposure to that speciality that might translate in to less consideration of that speciality as a career option after graduation [28].

Wrighton and Lawrence argued in the summer of 2020, that colleges and universities needed to re-open but that there was no risk-free way to do this and that there might not be a uniform approach [27]. We adopted a multimodal strategy including barrier-free and accessible COVID-19 testing, timely contact tracing, mandatory IPC procedures, safe in-person learning, clinical placements and simulation, all possible during a pandemic. Data from China earlier on in the pandemic reported high frequency of mask wearing, hand hygiene and working from home as much as possible, amongst medical students, despite the occurrence of anxiety and depression [29]. This is reassuring as it indicates that students will comply with measures to ensure their own and others’ safety during a crisis.

The interactions between students and educators is crucial. Indeed, the importance of experiencing and contributing to teamwork from a very early stage with inter-professional education is increasingly recognised because it reflects the increasing complexity of the care of many patients (e.g. diabetes mellitus) and because it contributes to safety in the workplace [30]. Interactions between medical students, their educators and medical doctors contributes to their professional identity formation and socialisation [31]. Socialisation includes a combination of existing personal identities, the influence of role models and mentors, and both formal and self-assessment, resulting in the student acquiring their professional identity [32]. However, much of this is restricted if medical education is largely online.

Although circumstances have improved, thus facilitating greater on-site face-to-face experiences, the medical educational community must reflect on and maximise what can be achieved safely on campus [32]. Medical student vaccination has assisted in this, as they are tomorrow’s doctors and the vaccination of healthcare workers and the residents of long-term care facilities, were prioritised [33]. Face-to-face interactions were achieved by engagement with students, and by addressing their wellbeing, underpinned by regular testing and tracing, perhaps through rapid and more frequent point-of-care screening and vaccination [34]. Indeed, medical students themselves understand the need for more education on IPC, including on the wearing of personal protective equipment, and direction on the sourcing of reliable information [35]. The introduction of PCR testing also, using saliva samples, after these were shown to have a high correlation with the results from NPS, facilitated testing and screening [18, 21]. All these measures helped ensure as normal a medical educational experience as possible resulting in the timely graduation of fully educated and competent medical doctors. They will also inform the delivery of medical education during any recurrence and subsequently during any new major transmissible infection emergency.

The limitations of what we describe include the fact that it was a single university/medical school experience, and the lack of a direct comparison group as any student comparisons were made between pre-and post-pandemic cohorts. While much of the data were collected prospectively, some were reviewed retrospectively, e.g. number of face-to-face teaching sessions. Furthermore, the interventions were reactive and not pre-determined given that they occurred during a pandemic, thus making it difficult to determine which aspects were most effective, and which were not so important.

## Conclusions

We have outlined the approach of one healthcare university in addressing the challenge of delivering a robust engaged in-person medical education programme throughout the COVID-19 pandemic, including maintaining clinical placements. This was possible because of clear leadership from the top and an effective, agile and responsive MDT that prioritised the student experience. This delivered the equivalent amount of face-to-face teaching to that which occurred before the pandemic. This was achieved by proactive two-way communication with students in which we addressed their health, emotional and social concerns. Other important components included the opening of an additional campus, testing for COVID-19 on-site, the early establishment of a vaccination programme and liaison with public health on contact tracing and related matters. While online education was important, efforts to optimise safe ongoing face-to-face medical education over an extended period of undergraduate education were seen as pivotal in forming the skills and professional identity of the next cohorts of early career medical graduates. The time and expense (€9.3m) were justified by the achievements. While our experience and setting may be different to other institutions elsewhere, our approach may inform and assist others when and if another pandemic or seriously disruptive event occurs.

## References

[1] Walsh J, Skally M, Duffy F, Kalukondanahally G, Dinesh B, O’Connell K, et al. The early test catches the case. Why wait? Frequent testing of close contacts aids COVID-19 control. J Hosp Infect 2021; 116:101–102. doi: 10.1016/j.jhin.2021.08.004 34403764 PMC8364144

[2] Abbas M, Nunes TR, Martischang R, Zingg W, Iten A, Oittet D, et al. Nosocomial transmission and outbreaks of coronavirus disease 2019: the need to protect patients and healthcare workers. Antimicrob Resist Infect Control 2021 10:7. 10.1186/s 13756-020-00875-7. 33407833 PMC7787623

[3] Bielicki BA, Duval X, Gobat N, Goossens H, Koopmans M, Tacconelli E, et al. Monitoring approaches for health-care workers during the COVID-19 pandemic. Lancet Infect Dis 2020;20 e261–267. doi: 10.1016/S1473-3099(20)30458-8 32711692 PMC7377794

[4] Daniel M, Gordon M, Patricio M, Hider A, Pawlik C, Bhagdev R, et al. An update on developments in medical education in response to the COVID-19 pandemic: A BME scoping review: BEME Guide No. 64. Med Teacher 2021 doi: 10.1080/0142159X.2020.1864310 33496628

[5] Sandhu P, de Wolf M. The impact of COVID-19 on the undergraduate medical curriculum. Med Educ Online 2020; 25: 1764740. doi: 10.1080/10872981.2020.1764740 32400298 PMC7269089

[6] Rose S. Medical student education in the time of COVID-19. JAMA 2020; 323: 2131–2132. doi: 10.1001/jama.2020.5227 32232420

[7] Gill D, Whitehead C, Wondimagegn D. Challenges to medical education at a time of physical distancing. Lancet 2020; 396: 77–79. doi: 10.1016/S0140-6736(20)31368-4 32534631 PMC7289574

[8] Choi B, Jegatheeswaran L, Minocha A, Alhilani M, Nakhoul M, Mutengesa E. The impact of the COVID-19 pandemic on final year medical students in the United Kingdom: a national survey. BMC Med Educ 2020; 20: 206. doi: 10.1186/s12909-020-02117-1 32600460 PMC7323883

[9] Pfefferbaum B, North CS. Mental health and the Covid-19 pandemic. New Engl J Med 2020; 383-510–512. doi: 10.1056/NEJMp2008017 32283003

[10] Karatzias T, Shevlin M, Murphy J, McBride O, Ben-Ezra M, Bentall RP, et al. Posttraumatic stress symptoms and associated comorbidity during the COVID-19 pandemic in Ireland: a population-based study. J Trauma Stress 2020: doi: 10.1002/jts.22565 32662129 PMC7405473

[11] Gallè F, Sabella EA, Da Molin G, De Giglio O, Caggiano G, Di Onofrio, et al. Understanding knowledge and behaviors related to CoViD-19 epidemic in Italian undergraduate students: The EPICO Study. Int J Environ Res Public Health 2020; 17: 3481; doi: 10.3390/ijerph17103481 32429432 PMC7277609

[12] European Centre for Disease Control. COVID-19 country overviews. https://covid19-country-overviews.ecdc.europa.eu/.

[13] Ethical framework for asymptomatic COVID-19 testing. Students in higher educational institutions. The Healthcare Improvement Studies Institute. University of Cambridge. February 2021 (https://www.thisinstitute.cam.ac.uk/research-articles/covid-19-ethical-framework-for-asymptomatic-testing-of-students-in-higher-education-institutions/).

[14] Leijon M, Lundgren B. Connecting physical and virtual spaces in a HyFlex pedagogic model with a focus on teacher interaction. J Learn Spaces 2019; 8: 1–9.

[15] Raes A, Detienne L, Windey I, Depaepe F. A systematic literature review on synchronous hybrid learning: gaps identified. Learn Environ Res 2020; 23: 269–290.

[16] Beatty BJ. Hybrid-Felxible Course Design. EDTech Books. https://edtechbooks.org/hyflex/.

[17] National Health and Safety Function, Information and Advisory Team. Health Service Executive, Ireland. COVID-19 Test Centre Checklist. January 2021. (https://healthservice.hse.ie/filelibrary/coronavirus/covid-19-test-centre-checklist.docx. Accessed 14-06-21).

[18] De Santi C, Jacob B, Kroich P, Doyle S, Ward R, Li B, et al. Concordance between PCR-based extraction-free saliva and nasopharyneal swabs for SARS-CoV-2 testing. HRB Open Res 15 October 2021, 4:85 doi: 10.12688/hrbopenres.13353.2 34522839 PMC8408542

[19] Lu X, Wang L, Sakthivel SK, Whitaker B, Murray J, Kamili S, et al. US CDC Real-time reverse transcription PCR panel for detection of severe acute respiratory syndrome Coronavirus 2. Emerg Infect Dis 2020; 26: 1654–1665. doi: 10.3201/eid2608.201246 32396505 PMC7392423

[20] Braun V., Clarke V. Using thematic analysis in psychology. Qual. Res. Psychol. 2006;3:77–101.

[21] DeSanti C, Cavalleri GL, Kerrigan SW, Fitzpatrick F, McGrogan K, Gilroy J, et al. Screening medical students for SARS-CoV-2 to facilitate face-to-face clinical teaching and prevent onward spread to patients. J Hosp Infect 2023;135: 1–3 (ahead of print). doi: 10.1016/j.jhin.2023.02.001 36775068 PMC9916130

[22] Luck J, Gosling N, Saour S. Undergraduate surgical education during COVID-19: could augmented reality provide a solution? Br J Surg 2021; 108: e129–130. doi: 10.1093/bjs/znaa159 33793706 PMC7929321

[23] Dow N, Wass V, MacLeod D, Muirhead L, McKeown J. ‘GP Live’- recorded General Practice consultations as a learning tool for junior medical students faced with the COVID-19 pandemic restrictions. Ed Primary Care 2020; 31(6): 377–381. doi: 10.1080/14739879.2020.1812440 32842902

[24] Shin TH, Kingler M, Han A, Mocsiran JL, Vilchez V, Naples R, et al. Efficacy of virtual case-based general surgery clerkship curriculum during COVID-19 distancing. Med Sci Ed 2020 doi: 10.1007/s40670-020-01126-5 33200037 PMC7654350

[25] Stout RC, Roberts S, Maxwell-Scott H, Gothard P. Necessity is the mother of invention: how the COVID-19 pandemic could change medical student placements for the better. Postgrad Med J 2021;): 1–6. doi: 10.1136/postgradmedj-2021-139728 33593809 PMC7887865

[26] Donohue KE, Farber DL, Goel N, Parrino GR, Retener NF, Rizvi S, et al. Quality improvement amid a global pandemic: a virtual curriculum for medical students in the time of COVID-19. MedEdPORTAL 2021;17.11090. doi: 10.15766/mep_2374-8265.11090 33598535 PMC7880258

[27] Wrighton MS, Lawrence SJ. Reopening colleges and universities during the COVID-19 pandemic. Ann Intern Med 2020; 173: 664–665. doi: 10.7326/M20-4752 32614640 PMC7339038

[28] Milligan F, Langhe R. COVID-19 and teaching challenges. Ir Med J 2020; 114: 254.

[29] Xiao H, Shu W, Li M, Li Z, Tao F, Wu X, et al. Social distancing among medical students during the 2019 Coronavirus disease pandemic in China: disease awareness, anxiety disorder, depression, and behavioural activities. Int J Envir Res Public Health 2020; 17, 5047; doi: 10.3390/ijerph17145047 32674285 PMC7399842

[30] Chandrashekar A, Mohan J. Preparing for the National Health Service: the importance of teamwork training in the United Kingdom medical school curriculum. Adv Med Ed Pract 2019; 10: 679–688. doi: 10.2147/AMEP.S203333 31686942 PMC6709809

[31] Cruess RL, Cruess SR, Boudreau JD, Snell L, Steinert Y. A schematic representation of the professional identity formation and socialization of medical students and residents: A guide for medical educators. Acad Med 2015; 90: 718–724. doi: 10.1097/ACM.0000000000000700 25785682

[32] Skegg D, Gluckman P, Boulton G, Hackmann H, Karim SSA, Piot P, et al. Future scenarios for the COVID-19 pandemic. Lancet 2021; 397: 777–778. doi: 10.1016/S0140-6736(21)00424-4 33607000 PMC7906624

[33] Jain V, Schwarz L, Lorgelly P. A rapid review of COVID-19 vaccination prioritisation in the US: alignment between federal guidance and state practice. Int J Envir Res Pub Health 2021 18, 3483. https://doi.org 10.3990/ijerph18073483. 10.3390/ijerph18073483 PMC8036633 33801651

[34] Cheap and quick: Could rapid antigen testing be the way out of lockdown? https://www.irishtimes.com/news/health/cheap-and-quick-could-rapid-antigen-testing-be-the-way-out-of-lockdown-1.4481373.

[35] Cheng C, O’Donnell S, Humphreys H. Medical education, the COVID-19 pandemic, and infection prevention: there has never been a better time. J Hosp Infect 2022; 119: 187–88. doi: 10.1016/j.jhin.2021.11.015 34848295 PMC8627300

